# Supplementary material for: A Summary of Practical Considerations for the Application of the Steric Exclusion Chromatography for the Purification of the Orf Viral Vector
Source: Membranes (Basel). 2022 Oct 29;12(11):1070. doi: 10.3390/membranes12111070 (PMC9696199; doi:10.3390/membranes12111070)
Supplement: Supplementary file 1 [file membranes-12-01070-s001.zip › membranes-1914572-supplementary.pdf]

Supplementary Material:

A summary of practical considerations for the application of the steric exclusion chromatography for the purification of the Orf viral vector

S1: Influence of salts on the infectivity assay 2

S2: Screening of relevant process parameters for SXC with the ORFV 4

S3: Description of the ORFV aggregation behaviour in dependence of PEG_{6000} and pH 6

S3.1 Statistical analysis of ORFV aggregation kinetics 6

S3.2 Visualization of cell culture-derived ORFV aggregates 9

S4: Chromatograms 10

S4.1 SXC with varying incubation times 10

S4.2 Application of different buffers 11

S4.3 Application of different salts 12

S5: pH-dependent charge measurements of ORFV with NaCl and $MgCl_2$ 12

References 14

S1: Influence of salts on the infectivity assay

The ORFV remained infectious in presence of all tested salts (KCl, NaCl, $NaNO_3$, $MgCl_2$, $(NH_4)_2SO_4$, Na_2SO_4, and $MgSO_4$) for the duration of the SXC experiments (data not shown), however, the influence of the salts on the cytometric infectivity assay itself was not assessed before.

Material and Methods

The influence of the implemented salts (KCl, NaCl, $NaNO_3$, $MgCl_2$, $(NH_4)_2SO_4$, Na_2SO_4, and $MgSO_4$) on the infectivity assay was assessed by the change of the infection result, i.e., percentage of virus positive cells compared to a positive control (100 %). For this purpose, Vero cells were prepared as described in **section 2.3** and each well was infected with 50 µL of virus stock (2×10^6 IU mL^{-1}). Immediately afterwards, 100 µL of salt solution was added. The salt solutions, with concentrations of 2 – 200 mM, were prepared in neutral 20 mM TRIS buffer. The positive control (PCTRL) was DMEM with 5 % FCS, known to stabilize the ORFV. Additionally, a negative control (NCTRL) of pure TRIS buffer without salt addition was titrated. Each salt concentration was tested in quadruplicates. The mean readout was normalized to the positive control (100 %). The statistical analysis was performed by ANOVA with a *Tukey* test ($\alpha = 0.05$) (Origin Pro 2021b, OriginLab Corporation).

Results and Discussion

The addition of salts to the infectivity assay indicated a small impact on the readout of infected cells for most tested concentrations (**Figure S1**). KCl, NaCl, $MgCl_2$, and Na_2SO_4 revealed no significant change for 50 – 200 mM. Concerning $NaNO_3$ and $MgSO_4$, only 50 mM and 200 mM affected the assay by lower relative readouts of 12 % and 31 %, respectively. As the addition of the salt-free buffer (NCTRL) revealed a 4 % relative reduction, we assumed that the impact of 50 mM $NaNO_3$ was still in the range of the relative error of the assay (up to 10 %). However, $MgSO_4$ should be diluted at least down to 100 mM to prevent deviating results. Concerning $(NH_4)_2SO_4$, the only salt tested in the full range, 2 – 200 mM, indicated a negative impact on cell viability at concentrations ≥5 mM. In this assay, a destabilizing effect on the virus itself could not be eliminated. Thus, $(NH_4)_2SO_4$ was excluded from further experiments.

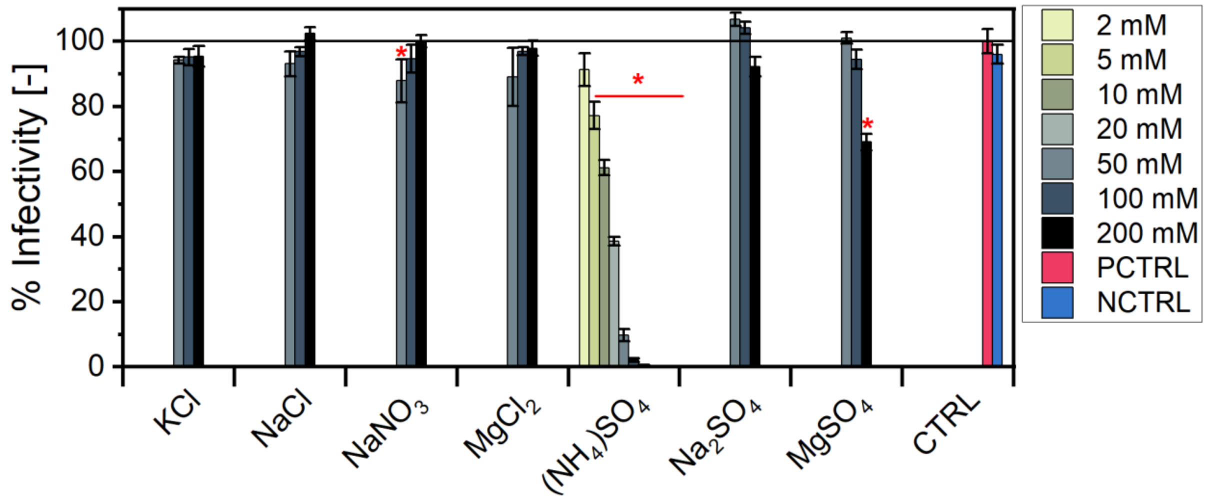

Figure S1: Influence of salts on the infection of Vero cells with the ORFV.

The impact of seven different salts (KCl, NaCl, $NaNO_3$, $MgCl_2$, $(NH_4)_2SO_4$, Na_2SO_4, and $MgSO_4$) on the readout of the infectivity assay with the ORFV was assessed. Therefore, cells were infected with ORFV, and immediately afterwards, salt solutions with concentrations of 2 – 200 mM were applied. After incubation, the percentage of virus-positive cells was measured, and compared to a positive control (PCTRL) (100 %), which consisted of DMEM with FCS. Additionally, a negative control (NCTRL) was prepared with pure TRIS buffer without salt addition. The deviation from the PCTRL was statistically analyzed by ANOVA with a *Tukey* test ($\alpha = 0.05$). Asterisks indicate significance.

S2: Screening of relevant process parameters for SXC with the ORFV

A DOE-based approach was chosen to evaluate the infectious ORFV yield in the SXC process, depending on the PEG concentration (4 – 8 %), the PEG molecular weight (6,000 – 12,000 Da), and the flow rate (0.5 – 6.0 mL min^{-1}). **Table S1** depicts the characteristics of the design, and **Table S2** the ANOVA for the response of infectious ORFV yield.

The resulting model for the response of the ORFV recovery in the elution fraction combines the three factors from **Table S2**. The prediction of the PEG concentration and molecular weight is depicted in **Figure 1A** in the main text. **Figure S2** shows the influence of the flow rate and the PEG precipitation on the SXC performance. A maximum of 60 % ORFV yield was achieved for high PEG concentrations (8 %) and low flow rates (0.5 mL min^{-1}). The recovery was reduced to 35 % for the other extrema of the two factors. Such dependencies of the target retention and the residence time in the SXC were reported before [1]. By reducing the flow rate, the chance of target accretion to the membrane, which is a random process governed by target-surface collisions, is increased. Additionally, shear forces by the passing fluid, which can reverse the accretion, are reduced [2].

Table S1: Model characteristics for the evaluation of the infectious ORFV yield in the SXC process with varying flow rates, PEG concentration, and molecular weight.

**Study Type**	Response surface
**Design Type**	I-optimal
**Subtype**	Randomized
**Runs**	24
**Blocks**	No

Table S2: ANOVA of the infectious ORFV yield in the SXC process with varying flow rates, PEG concentration, and molecular weight.

Clarified ORFV cell culture supernatant was processed via SXC with varying PEG concentration (4 – 8 %) (c_{PEG}), PEG molecular weight (MW) (6,000 – 12,000 Da), and flow rates (0.5 – 6.0 mL min^{-1}). The elution fraction of the SXC was analyzed regarding the infectious ORFV concentration and the yield calculated. The data was analysed by ANOVA (α = 0.05) for a linear model.

	Infectious ORFV yield [%]		
**Source**	**F-value**	**p-value**	
A (PEG MW)	2.67	0.1190	significant
B (% c_{PEG})	10.95	0.0037	significant
C (Flow rate)	10.64	0.0041	significant

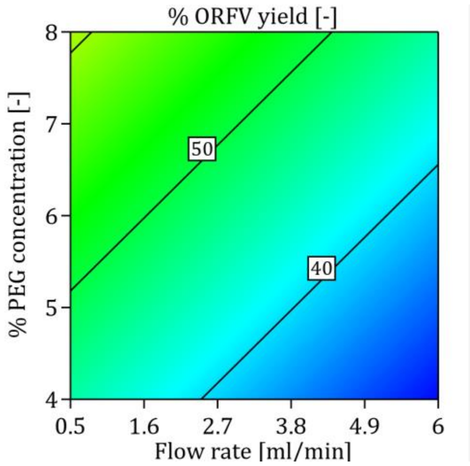

Figure S2: ORFV yield after SXC process with varying PEG concentration and flow rates

Clarified ORFV cell culture supernatant was processed via SXC with varying PEG concentration (4 – 8 %) and flow rate (0.5 – 6.0 mL min^{-1}). The PEG molecular weight was set to 6,000 Da. The elution fraction of the SXC was analyzed regarding the infectious ORFV concentration and the calculated yield. The colouring of the contour plots is coded as follows for the infectious ORFV yield: 30 – 40 % (blue), 40 – 45 % (light blue), and 45 – 60 % (green).

S3: Description of the ORFV aggregation behaviour in dependence of PEG_{6000} and pH

S3.1 Statistical analysis of ORFV aggregation kinetics

Aggregation kinetics, evaluating the influence of time, pH, and PEG_{6000} concentration on the size distribution of ORFV-containing samples, were conducted by dynamic light scattering measurements over the course of 60 min.

Table S3: Model characteristics for the evaluation of ORFV aggregation kinetics with varying pH and PEG concentrations

**Study Type**	Response surface
**Design Type**	Historical Data
**Subtype**	Split-plot
**Groups**	19
**Runs**	826
**Blocks**	No

Table S4: ANOVA of ORFV aggregation kinetics with varying pH and PEG concentrations.

Clarified ORFV cell culture supernatant was mixed 1:4 with concentrated buffers to generate defined pH (4 – 7.4) and PEG_{6000} (0 – 12 %) concentrations (c_{PEG}). The size distribution of the 19 different samples (groups) was measured every 5 min by DLS over the course of 60 min. The data was analyzed by a restricted maximum likelihood ANOVA ($\alpha = 0.05$) for a quadratic split-plot model. The whole-plot represents the unchanged factors (pH and PEG concentration) in each of the groups, and the subplot is used to display the time-dependent changes within.

	**Size [nm]**		
**Source**	**F-value**	**p-value**	
Whole-plot	94.08	< 0.0001	significant
a (pH)	27.00	0.0002	significant
b (% c_{PEG})	429.85	< 0.0001	significant
ab	0.28	0.6088	
a^2	4.45	0.0549	
b^2	16.21	0.0015	significant
Subplot	489.33	< 0.0001	significant
C (time)	92.83	< 0.0001	significant
aC	1.33	0.2487	
bC	225.56	< 0.0001	significant
C^2	140.47	< 0.0001	significant

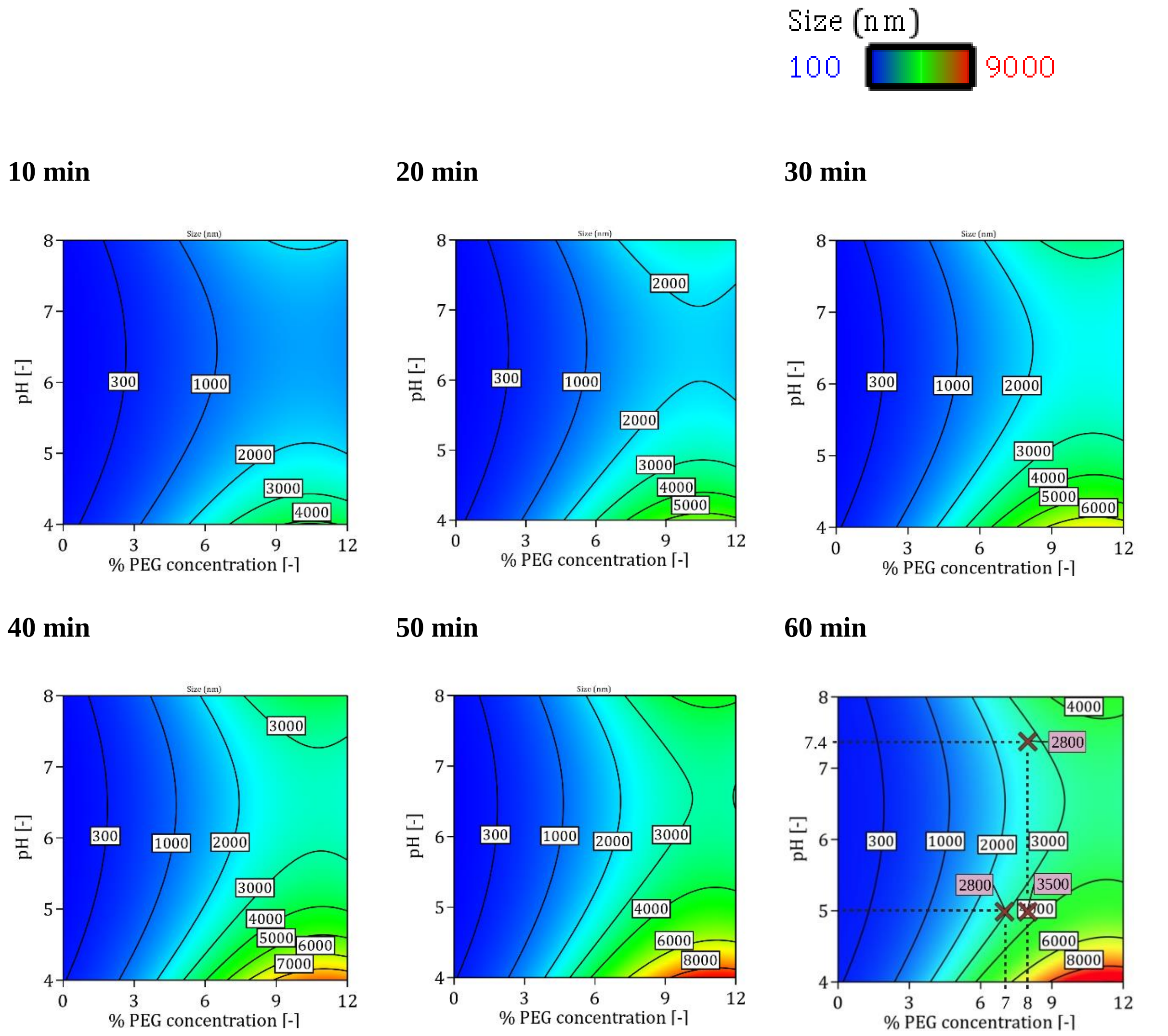

Figure S3: pH-dependent aggregation of cell culture-derived Orf viruses (ORFV) in the presence of polyethylene glycol (PEG).

Aggregation kinetics of the ORFV were analyzed using dynamic light scattering in dependence of the pH (4 – 7.4) and the PEG_{6000} (6,000 Da) concentration (0 – 12 %), mimicking conditions of the steric exclusion chromatography. Over the course of 60 min, measurements were conducted automatically every 5 min on each sample. After each incubation, the respective sample was visualized by bright-field microscopy (**Figure S4)**. Using a design of experiments-based approach, the size response data was statistically analyzed (**Table S4**) and a model was generated. The colouring of the contour plots is coded as follows for the mean size: 100 – 2,000 nm (blue), 2,000 – 3,000 nm (light blue), 3,000 – 6,000 nm (green), 6,000 – 7,000 nm (yellow), and 7,000 – 9,000 nm (red).

Additional attributes in the statistical analysis were the low variance between the groups (0.125), which rendered the analysis equivalent to a randomized design, as well as the overall small variance (0.145),

the high R^2 (0.99), and the minimal difference between R^2 and the adjusted R^2 ($\Delta = 0.0002$), which indicated a well-fitted model.

The model predicts the design space for the factors PEG_{6000} concentration and pH values, visualized in **Figure S3**. Observing the impact of the pH on the aggregation behavior, a maximum in precipitate size was reached at the highest tested PEG concentration (12 %) and the lowest pH value (pH 4). This pH value is in close proximity of the isoelectric point determined for the applied ORFV genotype in CPB of pH 3.5 (data not shown). At pH 4, omitting PEG, no spontaneous aggregation was observed by DLS. However, an increase of self-association tendencies has to be expected [3].

S3.2 Visualization of cell culture-derived ORFV aggregates

After the dynamic light scattering measurements, the samples of each run were visualized by bright-field microscopy in a conventional Neubauer chamber in order to control the observed volume. **Figure S4** depicts four of the extrema representing the conducted runs.

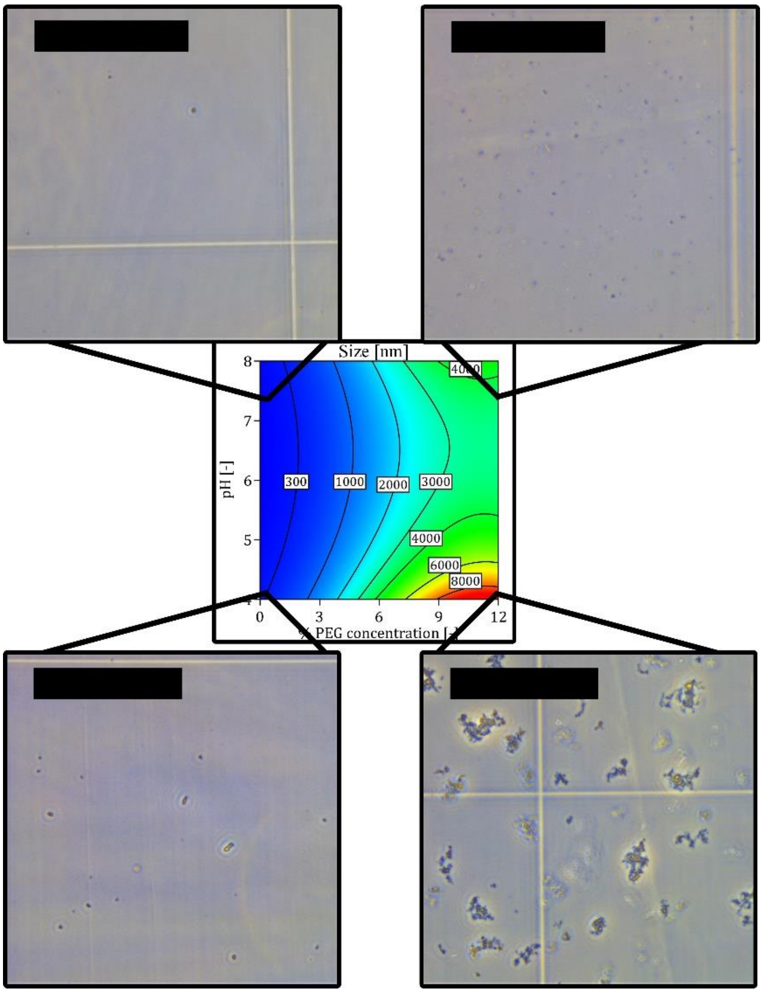

Figure S4: Visualization of the influence of the PEG_{6000} concentration and the pH on the ORFV aggregation size.

Clarified ORFV cell culture supernatant was combined with CPB according to a DOE-based plan. The final mixes of deviating pH (4 – 7.4) and PEG_{6000} concentrations (0 – 12 %) were analyzed using dynamic light scattering over the course of 60 min. The kinetic data was statistically analyzed (**Table S4**), and a model was generated of the size response (**Figure S3**). After each measurement run, a defined volume of the sample was visualized by bright-field microscopy in a Neubauer chamber. Here, the contour plot for 60 min incubation time is presented again to facilitate a comparison, augmented by the micrographs of the model's corner points. The bar corresponds to 100 µm.

S4: Chromatograms

S4.1 SXC with varying incubation times

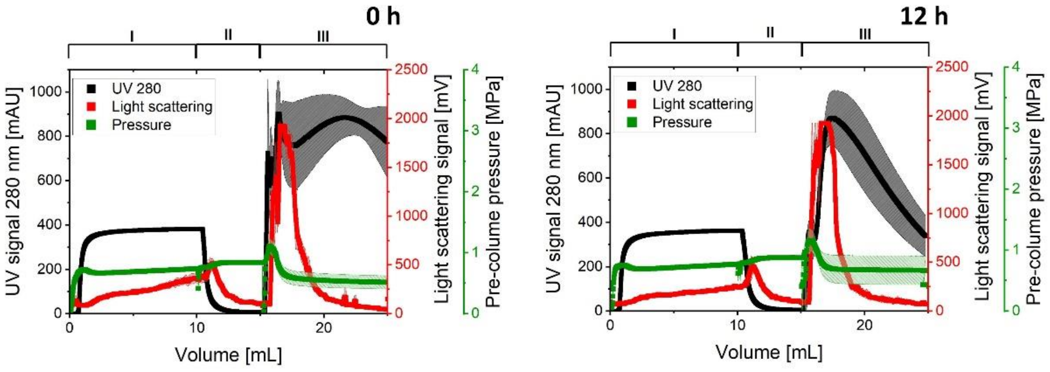

Figure S5: Chromatograms of SXC applications of the ORFV with varying incubation times

The Orf virus (ORFV) was processed via SXC (load: 8 % PEG_{8000} (polyethylene glycol, 8,000 Da); elution: 0 % PEG, 0.4 M NaCl) in citrate phosphate buffer with a pH-value of 7.4. The pre-mixed ORFV/PEG solution was either directly applied to the column (0 h), or incubated for 12 h before loading. The triplicate runs were fractionated into flow-through, i.e., sample application (I), wash (II), and elution (III). The online monitoring included an UV signal at 280 nm (black), light scattering signal (red), and the pre-column pressure (green). The hatched areas indicate the respective standard deviations.

S4.2 Application of different buffers

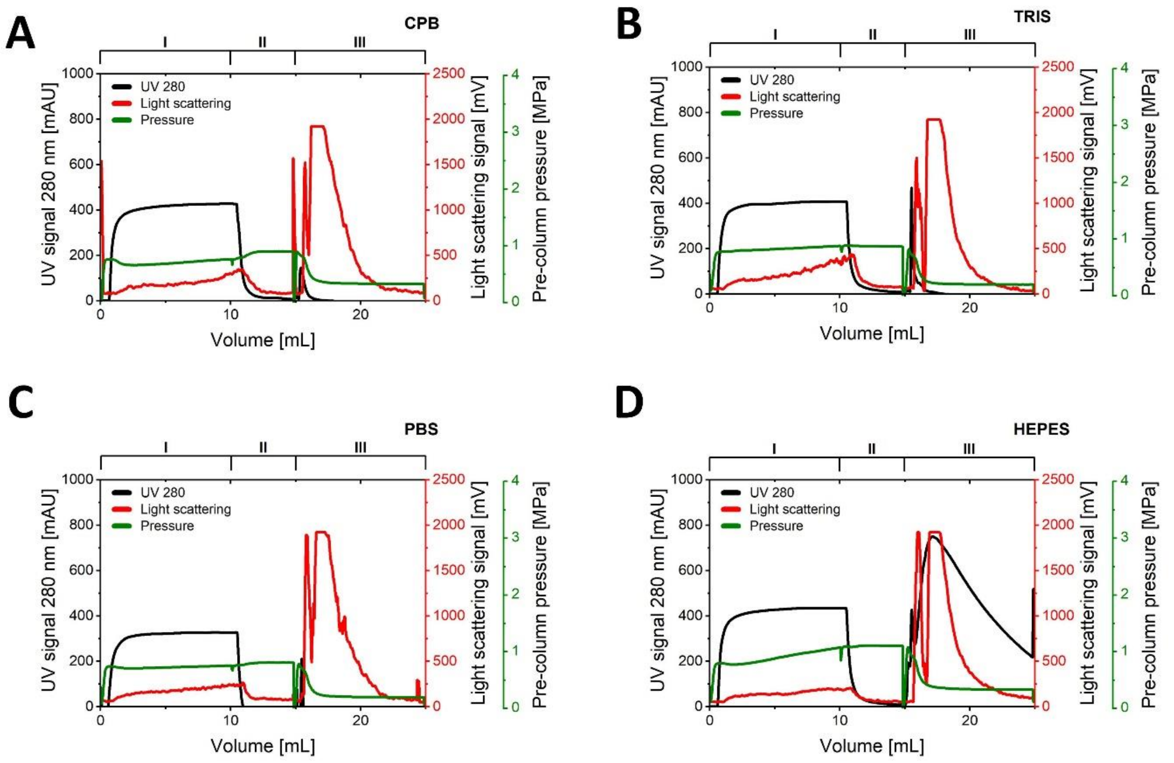

Figure S6: Chromatograms of SXC applications of the ORFV with different buffers

The ORFV was processed via SXC in different buffering systems: 0.1 M citrate phosphate buffer (CPB, **A**), 20 mM TRIS-HCl (**B**), PBS (**C**), and 0.1 M HEPES (**D**). All buffers were of neutral pH and adjusted to a conductivity of 15 mS cm^{-1} with NaCl. Each buffering system was used for all process steps, i.e., the load and wash buffer (8 % PEG_{8000}) as well as the elution buffer (0 % PEG, 0.4 M NaCl). The runs were fractionated into flow-through, i.e., sample application (I), wash (II), and elution (III).

S4.3 Application of different salts

Table S5: Summary of load and pre-column pressure of SXC applications of the ORFV with different salts

Concentration	Salt	Mean load [mL]	STD	Mean pre-C pressure [MPa]	STD
200 mM	NaCl	31.7	1.2	0.40	0.00
50 mM	NaCl	38.0	1.3	0.40	0.00
20 mM	NaCl	29.2	8.2	0.38	0.03
	KCl	36.2	5.3	0.37	0.02
	$NaNO_3$	28.2	3.5	0.40	0.00
	$MgCl_2$	39.5	0.8	0.38	0.01
	Na_2SO_4	31.4	4.0	0.40	0.00
	$MgSO_4$	31.8	5.8	0.38	0.04
	$(NH_4)_2SO_4$	27.8	0.1	0.40	0.00

S5: pH-dependent charge measurements of ORFV with NaCl and $MgCl_2$

We tested the impact of the protein-salt interaction by monitoring the pH-dependent zeta potential of the ORFV in the presence of different salt concentrations, under the omission of PEG (**Figure S7**). At 15 mS cm^{-1}, the pH-dependent charge was a similar function for both salts ranging from -6 mV at pH 4.5 to the isoelectric point at pH 3.5. $MgCl_2$ reduced the charge at pH 2.5 by roughly 1 mV compared to NaCl, and lead to a steeper y-intercept passage. With increasing conductivity, the pH of the isoelectric point was reduced to approximately pH 3 at 35 mS cm^{-1} for both salts. Here, the function for NaCl was roughly linear, still down to -6 mV at pH 4.5. $MgCl_2$, on the contrary, diminished the charge to -3 mV at the latter pH, and the charge remained constant (0 mV) at pH values below the isoelectric point. At 45 mS cm^{-1}, the picture was similar to that for 45 mS cm^{-1} for NaCl, with an isoelectric point at pH 2.5. For $MgCl_2$, however, no positive charge was measured at this concentration. This observation implies that charge shielding was more effective for Mg^{2+} than for Na^+, as was reported before for divalent ions [4]. Charge shielding increases the efficiency of SXC, as it facilitates target association. This was not observed in the ORFV precipitation kinetics (**Figure 4D+E**). Thus, the effect of charge-shielding was neglectable for the applied salt concentration range. Interestingly, both salts showed specific interactions with the virus surface molecules, demonstrated by the change of the isoelectric point with increasing salt concentrations.

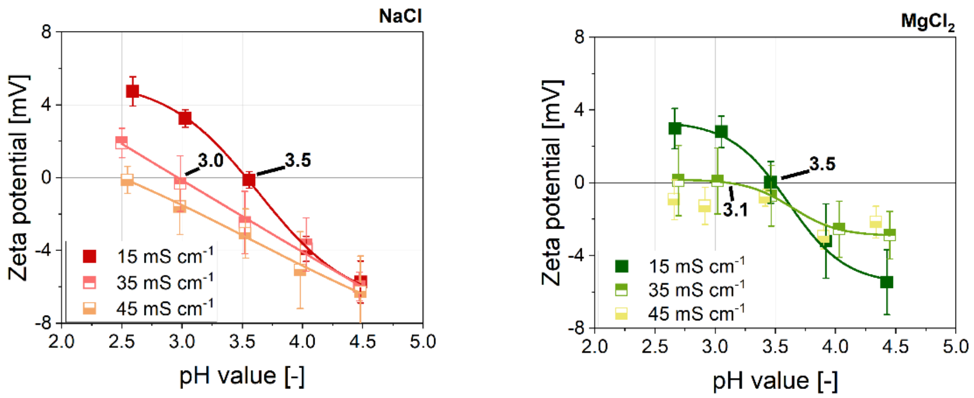

Figure S7: pH-dependent surface charge of the Orf virus (ORFV).

Purified ORFV was titrated to a range of pH values (2.5 – 4.5) with a citrate phosphate buffer, covering the isoelectric point of the virus. The samples were spiked with NaCl (left) and $MgCl_2$ (right) to equal 15, 35, or 45 mS cm^{-1}.

References

[1] S.-P. Tao, J. Zheng, Y. Sun, Grafting Zwitterionic Polymer onto Cryogel Surface Enhances Protein Retention in Steric Exclusion Chromatography on Cryogel Monolith, J Chromatogr A 1389 (2015) 104–111. https://doi.org/10.1016/j.chroma.2015.02.051.

[2] J. Lee, H.T. Gan, S.M.A. Latiff, C. Chuah, W.Y. Lee, Y.-S. Yang, B. Loo, S.K. Ng, P. Gagnon, Principles and Applications of Steric Exclusion Chromatography, J Chromatogr A 1270 (2012) 162–170. https://doi.org/10.1016/j.chroma.2012.10.062.

[3] S.I. Miekka, K.C. Ingham, Influence of self-association of proteins on their precipitation by poly(ethylene glycol), Arch Biochem Biophys 191 (1978) 525–536. https://doi.org/10.1016/0003-9861(78)90391-0.

[4] S. Salgyn, U. Salgyn, S. Bahadir, Zeta Potentials and Isoelectric Points of Biomolecules: The Effects of Ion Types and Ionic Strengths, Int. J. Electrochem. Sci. 7 (2012) 12404–12414.
